# Primary sternal osteomyelitis in a 40 days old infant: a case report and review of the literature

**DOI:** 10.4076/1757-1626-2-7504

**Published:** 2009-06-05

**Authors:** Nikolaos S Pettas, Alexandros P Apostolopoulos, Ioannis Flieger, Omiros Leonidou

**Affiliations:** 1 1^st^ Orthopaedic Department, Agia Sofia Children's HospitalBakogianni 104 B Street, Vrilissia Attikis, AthensGreece; 2 1^st^ Orthopaedic Department, Agia Sofia Children's Hospital99 Kyprou Street, Glyfada 16674, AthensGreece; 3 1^st^ Orthopaedic Department, Agia Sofia Children's HospitalKastalias 15 Street, Ekali 14578, AthensGreece; 4 1^st^ Orthopaedic Department, Agia Sofia Children's HospitalTheodosiou Sp. 34 Street, Chalandri 15234, AthensGreece

## Abstract

**Introduction:**

Primary sternal osteomyelitis is extremely rare in children and only very few cases have been reported in the international literature.

**Case presentation:**

A 40 days old Caucasian infant was referred to our clinic with a 4 days history of fever and malaise, accompanying a painful swelling of four days duration involving the lower end of the sternum. Examination revealed a 2 cm swelling which was fixed to the underlying bone. A full blood count, erythrocyte sedimentation rate, and C-reactive protein were measured and x-rays (Anterior and Lateral views) and Ultrasound was performed. Blood cultures were also taken. The patient was commenced empirically to Vancomycin and Cefotaxime intravenously.

The values of White Blood Cell (16,720), erythrocyte sedimentation rate (132 mm) and C-reactive protein (108 mg/dl) were elevated, the X-rays showed bone destruction and dislocation of the 3^rd^ sternal nuclei and in the U/S performed appeared a soft tissue mass measuring 2,37/1,02 cm related to the periosteum. Surgical debridement was then performed and swab cultures were taken intraoperatively. The infant grew *Streptococcus Pneumoniae* and *Enterococcus Species*. The infant was discharged after 25 days from the hospital. He gradually improved over a period of 2 months, became pain free and repeated x-rays showed significant bone resolution.

**Conclusion:**

Primary osteomyelitis in infants is a very rare condition that usually resolves with antibiotic therapy and surgical debridement.

## Introduction

Primary sternal osteomyelitis in infants is extremely rare. Very few cases have been reported in the International literature. In adults it usually occurs secondary to an underlying predisposition, such as immunodeficiency, IV drug abuse, Acne Fulminans, planoplantar pustulosis, cardiothoracic surgery or sternotomy [1-4]. In this review a case report of a 40 days old female infant is reported and its diagnosis and management are discussed.

## Case presentation

A 40 days old Caucasian infant was referred to our clinic with a four-day history of fever and malaise, accompanying a painful swelling of four days duration involving the lower end of the sternum. The infant was delivered with a normal labour and no incubator was required. The infant was initially admitted to the hospital in the pediatrics department due to fever (maximum temperature 39.8°C), and rhinitis was initially diagnosed. Nine hours after her admittance she was referred to our clinic and the clinical examination revealed a 2 cm swelling which was fixed to the underlying bone. A full blood count, ESR, CRP were measured and x-rays (Anterior and Lateral views) and Ultrasound was performed. Blood cultures were also taken. It is well proved that empiric antimicrobial therapy should cover Gram positive organisms and should be commenced once aspirate for cultures have been obtained. The patient was commenced to Vancomycin and Cefotaxime i.v. and was commenced after swab cultures were obtained.

**Figure 1.**
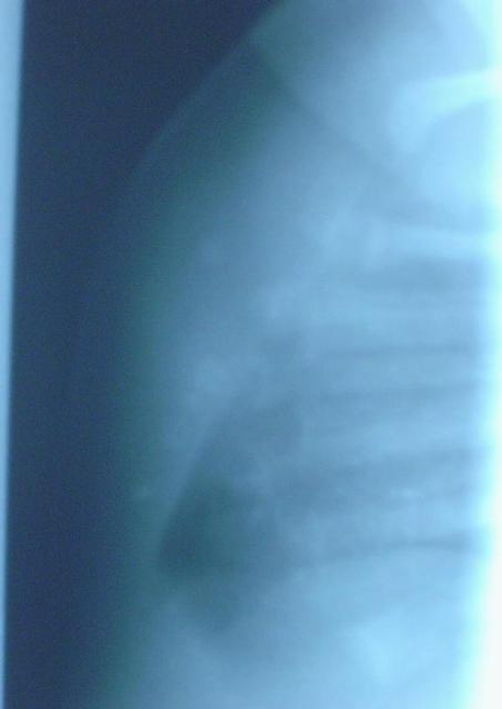
X-Ray of the sternum on day 1. Bone destruction and dislocation of the 3^rd^ sternal nuclei appear.

**Figure 2.**
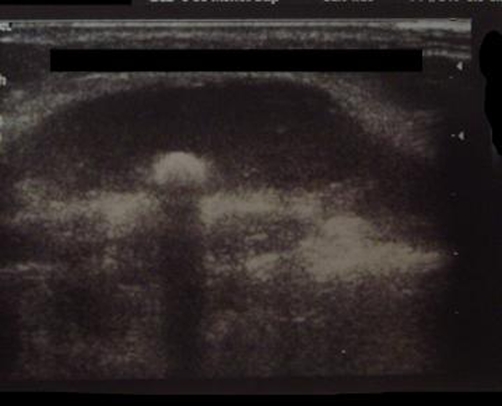
Ultrasound performed on day 1. Dislocation of the 3^rd^ sternal nuclei appear.

The values of WBC (17,620), ESR (132 mm) and CRP (108 mg/dl) were elevated, the X-rays showed bone destruction and dislocation of the 3rd sternal nuclei (Figure 1) and in the US performed appeared a soft tissue mass measuring 2.37 x 1.02 cm related to the periosteum (Figure 2). A diagnosis of osteomyelitis was made. The child was prepared for elective exploration and drainage.

At operation, the mass was approached through a longitudinal midline incision. A tense collection of pus was found surrounded by a thin membrane. The membrane was excised in its entirety and a small amount of the underlying bone, which appeared abnormal to the naked eye was removed with a curette. Surgical debridement was then performed and swab cultures were taken intraoperatively. The infant grew *Streptococcus pneumoniae* and *Enterococcus Species*.

**Figure 3.**
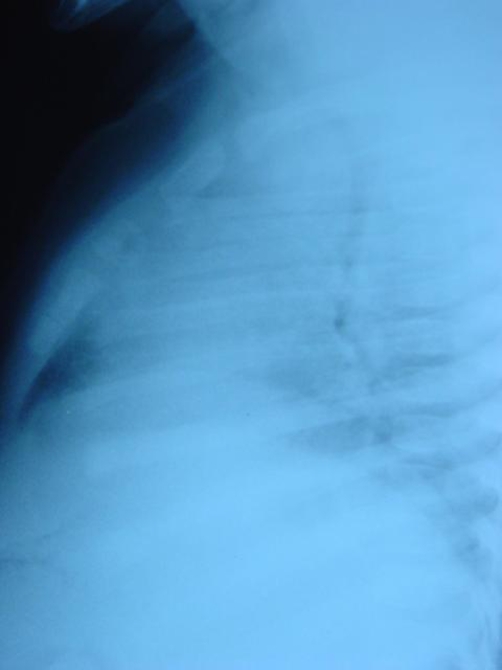
X-Ray performed during a follow up visit after 1 year.

**Figure 4.**
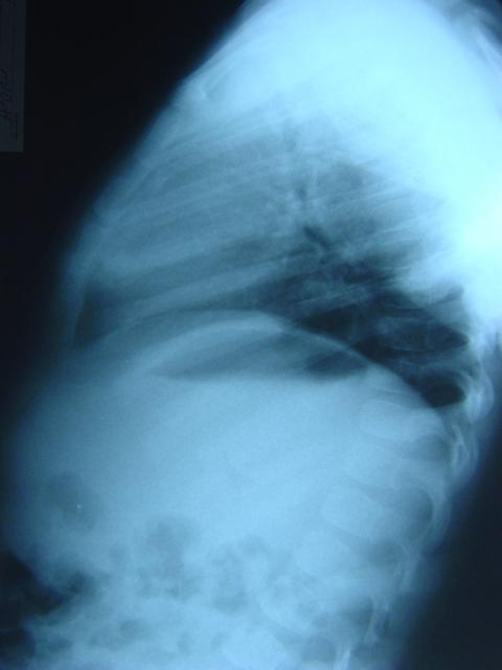
Complete bone resolution achieved 2 years later.

The infant was discharged after 25 days from the hospital. She gradually improved over a period of 2 months and became pain free. The infant was reexamined 2 months, 1 year and 2 years after her hospitalization in the outpatients department and repeated X-Rays showed complete bone resolution (Figures 3 & 4).

## Discussion

Sternal osteomyelitis is classified as primary when there is no other focus of infection and secondary when it occurs as a complication of median sternotomy, chest trauma, mediastinitis or subclavian intravenous line insertion. Primary sternal osteomyelitis in infants is extremely rare. Only very few cases of primary sternal osteomyelitis in infants have been reported in the international literature. Of these, 5 children had sickle cell disease and the causative organism was *Salmonella typhi* [5].

Although sternal swelling is uncommon in children primary sternal osteomyelitis should be considered in the differential diagnosis.

As with osteomyelitis at other sites, plain radiographs are often normal initially becoming abnormal after 2 weeks [6]. However, our patient had significant radiological findings soon after the onset of symptoms, suggesting that the disease process may have commenced without obvious clinical signs. Ultrasound is helpful in defining soft tissue collections and periosteal involvement, and technetium bone scan may localize the disease and exclude other foci of infection. CT scan and MRI are both useful in delineating the extent of the bony destruction and retrosternal or mediastinal collections. MRI is considered to be superior to CT scan [7]. Computed tomography is also helpful to guide accurate biopsy for microbiological and histopathologic diagnosis. Bone scanning can also aid in the diagnosis of osteomyelitis and in some cases it may show changes earlier than X-rays [4].

Tissue biopsy is essential in order to exclude primary bone pathology and to obtain a microbiological isolate that will direct appropriate antimicrobial therapy. Indirect measures such as CRP, WBC, and antistaphylosin titers may assist the diagnosis [8]. Empiric antimicrobial therapy should cover Gram positive organisms and should be commenced once aspirate for cultures have been obtained. Antimicrobials may be rationalized once identification and sensitivities are known. In our case the infant was commenced with Vancomycin and Cefotaxime after swab cultures were obtained for 14 days and continued with Cefotaxime for 10 more days. After her discharge from the hospital the i.v. therapy was switched to 14 days of oral animicrobials (Cefuroxime axetil). The average antibiotic treatment time suggested by most authors for the management of acute osteomyelitis in children is two weeks by intravenous (i.v.) administration followed by additional outpatient oral therapy for periods of up to four weeks. This treatment regimen applied specifically to acute osteomyelitis led to no known treatment failures. However, recent studies suggest that shorter courses of parenetral antibiotic therapy do appear to influence response rates for children with acute haematogenous osteomyelitis [9]. Le Chaux et al., conducted a systematic review of a short versus long course of treatment for Acute Haematogenous Osteomyelitis due primarily to *Staphylococcus aureus* in children aged 3 months to 16 years. They searched Medline, Embase and the Cochrane trials registry for controlled trials. Clinical cure rate at 6 months was the primary outcome variable, and groups receiving less than seven days of intravenous therapy were compared with groups receiving one week or longer of intravenous antimicrobials. The overall cure rate at six months for the short course of intravenous therapy was 95.2% (95% CI = 90.4 - 97.7) compared to 98.8% (95% CI = 93.6, 99.8) for the longer course of therapy. There was no significant difference in the duration of oral therapy between the two groups. Given the potential increased morbidity and cost associated with longer courses of intravenous therapy, this finding should be confirmed through a randomized controlled equivalence trial [9].

The most common infecting organism in both primary and secondary sternal osteomyelitis is *Staphylococcus aureus* [3,4,10] and *Pseudomonas aeruginosa* is the most common infecting organism in intravenous drug abusers [3,4,11]. Mycobacterium tuberculosis in endemic areas [12], *Aspergillus fumigatus* in immunocompromised patients [13] and *Candida albicans* [14] have also been reported to cause sternal osteomyelitis. In our case report apart of *Streptococcus pneumoniae*, *Enterococcus Species* was also isolated, an organism that has never been reported before as an infecting cause of primary sternal osteomyelitis in the international literature. In all cases of primary sternal osteomyelitis, bacterial spread is likely to be haematogenous [2]. The extreme porous nature of the sternum with its extensive Volkman canals and Haversian system, together with few reticuloendothelial cells and abundant bone marrow, may make the sternum susceptible to primary osteomyelitis [5].

Surgical debridement is usually necessary in severe cases of primary sternal osteomyelitis although the role of surgery is much more clearly established in osteomyelitis secondary to sternotomy or in the debilitated patient [2]. Vacuum assisted suction drainage also has proved to be a useful adjunct to surgical debridement [15].

In conclusion, primary sternal osteomyelitis in infants and children is very rare. Sternal pain and swelling are usually but not invariably present and the usual peripheral markers of inflammation are often insensible. This can lead to delayed diagnosis and inappropriate initial treatment. If left untreated the condition can lead to mediastinitis, chronic osteomyelitis and chest wall deformity and instability [4]. If it is suspected on clinical grounds, antibiotic therapy should be given immediately before awaiting the results of any investigations [4]: antistaphylococcal medication is the therapy of choice until further results of culture and sensitivity tests become available [3,10]. The optimal approach in uncomplicated cases may be a combination of aspiration for diagnostic purposes and prolonged antibiotic therapy. A patient's lack of response to antibiotic treatment or evidence of aggressive radiologic features are indications for surgery [3,4]. Any sternal swelling should be fully investigated radiologically and preoperatively to exclude malignancy [3,4]. Complete resolution should be expected [4].

## List of abbreviations

CRP: C-Reactive Protein
CT: Computed tomography
ESR: Erythrocyte sedimentation rate
US: Ultrasound
WBC: White blood cells
